# Breast cancer risk factors and mammographic density among high-risk women in urban China

**DOI:** 10.1038/s41523-018-0055-9

**Published:** 2018-02-06

**Authors:** Hyuna Sung, Jiansong Ren, Jing Li, Ruth M. Pfeiffer, Yong Wang, Jennifer L. Guida, Yi Fang, Jufang Shi, Kai Zhang, Ni Li, Shen Wang, Luopei Wei, Nan Hu, Gretchen L. Gierach, Min Dai, Xiaohong R. Yang, Jie He

**Affiliations:** 1 Division of Cancer Epidemiology & Genetics, National Cancer Institute, National Institutes of Health, Bethesda, MD 20850 USA; 2 National Cancer Center/Cancer Hospital, Chinese Academy of Medical Sciences and Peking Union Medical College, Beijing, 100021 China; 3 Department of Epidemiology, School of Public Health, China Medical University, Beijing, China

## Abstract

Elevated mammographic density (MD) is an established breast cancer risk factor. Studies examining relationships between MD and breast cancer risk factors are limited in China, where established breast cancer risk factors are less prevalent but dense breasts are more prevalent than Western countries. This study included 11,478 women (45-69 years; 36% premenopausal) participating in an ongoing national cancer screening program in 11 urban provinces in China and predicted as having high-risk for breast cancer. Polytomous logistic regression was performed to assess associations between MD and risk factors by comparing each higher Breast Imaging Reporting and Data System (BI-RADS) category (2, 3, or 4) to the lowest category (BI-RADS, 1). We found associations of increasing age, body mass index, weight, postmenopausal status, and parity with lower MD. Higher levels of education, increasing height, and later first birth were associated with higher MD. These associations did not vary by menopausal status. Additionally, the association between longer period of breastfeeding and lower MD was seen among postmenopausal women only (*P*_interaction_ = 0.003). Having first-degree relatives with breast cancer diagnosed before 50 years was associated with lower MD only among premenopausal women (*P*_interaction_ = 0.061). We found effects of established breast cancer risk factors on MD showed similar directions in Chinese and Western women, supporting the hypothesis that MD represents cumulative exposure to breast cancer risk factors over the life course. Our findings help to understand the biological basis of the association of MD with breast cancer risk and have implications for breast cancer prevention research in China.

## Epidemiology: Breast cancer risk factors similar in urban China and Western countries

Demographic variables linked to dense breast tissue — a strong risk factor for cancer — are similar among women living in China and the West. Xiaohong Yang from the US National Cancer Institute in Bethesda, Maryland, USA, and colleagues looked for predictors of elevated breast density, as measured on a mammogram, among participants of an ongoing cancer screening program in 11 urban provinces in China. In their analysis of 11,478 women aged 45 to 69 deemed at high-risk for breast cancer, the researchers identified many variables — incliuding age, height, weight, menopausal status, educational level and history of pregnancies — that were all related to mammographic density in some way. Similar associations were previously found among women in Western countries, suggesting that many of these risk factors are consistent across populations.

## Introduction

The estimated age-standardized incidence rate of breast cancer in China (22.1 per 100,000 person-years) is currently lower compared with Western countries (e.g., 92 per 100,000 person-years in Northern America).^1^ However, incidence rates have increased rapidly during the past decades and breast cancer is now the most frequently diagnosed female cancer in Chinese and Asian women.^2,3^ Previous studies have suggested that rising breast cancer incidence in Asian women is largely attributable to changes in reproductive and lifestyle risk factors such as declining fertility rates, delayed age at first birth, reduced breastfeeding, and increasing body mass index (BMI).^4–7^

Mammographic density (MD) reflects the proportion of radiologically dense epithelial and stromal breast tissues that are visible on a mammogram. The association between higher MD and breast cancer risk has been reported in diverse populations.^8–16^ However, in China, where the mammogram screening program is available only in selected areas and populations,^17^ the evaluation of MD in relation to breast cancer risk is very limited. Several breast cancer risk factors including age, reproductive factors, body size, menopausal hormonal therapy, and dietary factors are associated with variations in MD.^18–21^ This research suggests these risk factors can influence changes in breast tissue, manifested as variations in MD. Well-known risk factors for breast cancer explain 20–30% of the variance in percent mammographic density (PMD) in Western populations,^19,22^ suggesting that additional factors influencing MD variation remain to be identified. Knowledge of the genetic basis of MD is limited, but twin studies suggest that MD is a highly heritable trait and the majority (60–70%) of the residual MD variance can be explained by genetic factors after accounting for known MD influencing factors.^23^ Several studies show ethnic variations in MD and indicate that ethnicity is one of the factors that influences MD and may influence some of the associations between MD and breast cancer risk factors.^24–28^

Accordingly, the expansion of MD studies to populations with distinct genomic architecture and environmental and lifestyle exposures may improve our understanding of factors that influence MD and have important public health implications. Compared to Western populations, Asians are known to have higher PMD but lower prevalence of well-established breast cancer risk factors associated with Western lifestyle. Therefore, MD-influencing factors might not have exactly same effects in Asians as in Western populations. In addition, it is unknown how exposures with higher prevalence among Asian women such as tea drinking would influence MD.

Prior studies evaluating MD influencing factors among Asian women were limited to immigrant Asians^29,30^ or more developed Asian countries, such as Singapore,^31–34^ Japan,^35,36^ and Korea.^37–40^ Except for one large population-based study,^41^ MD studies conducted in China have been small and examined only a limited number of risk factors.^42^ Because density decreases with menopause,^43,44^ it is important to evaluate risk factor associations separately for premenopausal and postmenopausal women. However, none of these studies evaluated factors that influence MD by menopausal status.^41,42^ Therefore, we comprehensively examined the associations of MD with reproductive and lifestyle factors considering menopausal status among Chinese women who participated in a large multi-city screening program in China and had high predicted risk of developing breast cancer.

## Results

**Fig. 1:**
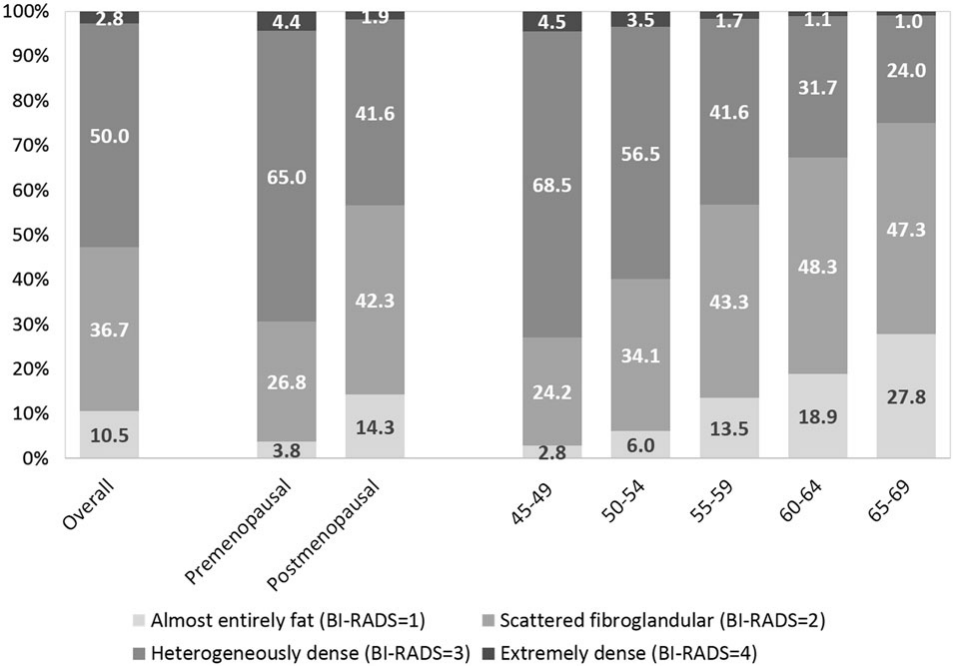
Distribution of mammographic density according to BI-RADS density categories by age and menopausal status

The distributions of selected characteristics are shown in Table 1. The mean age was 54.4 years (range, 45-69) and mean BMI was 24.1 kg/m^2^ (range, 15.8-36.4). Thirty percent of participants had first-degree relatives with breast cancer diagnosed before age 50. Most women were postmenopausal (64%), parous (94%), and reported that they had breastfed (83%), and had never smoked (87%) or drank alcohol (74%). The distributions of MD within Breast Imaging Reporting and Data System (BI-RADS) categories are shown for different age groups in Fig. 1. The overall distribution was as follows: 11% (1, almost entirely fat), 37% (2, scattered fibroglandular densities), 50% (3, heterogeneously dense), and 3% (4, extremely dense). Heterogeneously dense was the most common MD category for both premenopausal (65%) and postmenopausal (42%) women. As expected, MD decreased with increasing age (*P*_Mantel–Haenszel Chi-Square_ < 1.0E-30). MD also varied by family history, education, BMI, weight, age at menarche, parity, age at first full-term birth, breastfeeding, menopausal status, age at menopause, smoking, alcohol, and tea drinking (*P*_Chi-square or ANOVA_* < *0.05, Table 1).

**Table 1:** Demographic characteristics, anthropometric measures, reproductive and lifestyle factors among participants of the Chinese Breast Cancer Screening Program (2013–2014)

		All (*n* = 11478)		BI-RADS = 1 (*n* = 1206)		BI-RADS = 2 (*n* = 4216)		BI-RADS = 3 (*n* = 5736)		BI-RADS = 4 (*n* = 320)		*P* ^a^
		*n*	%	*n*	%	*n*	%	*n*	%	*n*	*%*	
Age												
Mean (SD)		54.4	6.3	59.0	5.96	55.9	6.20	52.5	5.70	51.1	5.22	<1.0E-30
45-49		3016	26.3	84	7.0	729	17.3	2066	36.0	137	42.8	<1.0E-30
50–59		5760	50.2	534	44.3	2191	52.0	2881	50.2	154	48.1	
60–69		2702	23.5	588	48.8	1296	30.7	789	13.8	29	9.1	
Family history of breast and/or ovarian cancers												
No		5797	50.5	581	48.2	2109	50.0	2939	51.3	168	52.7	0.05
1st or 2nd degree relatives^b^		2205	19.2	226	18.7	814	19.3	1092	19.0	73	22.9	
1st degree relatives with breast cancer <50 years		3472	30.3	399	33.1	1292	30.7	1703	29.7	78	24.5	
Missing		4				1		2		1		
Education												
None-elementary		1369	11.9	325	26.9	564	13.4	460	8.0	20	6.3	<1.0E-30
Middle school		3405	29.7	402	33.3	1321	31.3	1615	28.2	67	20.9	
High school		4054	35.3	336	27.9	1510	35.8	2083	36.3	125	39.1	
College+		2650	23.1	143	11.9	821	19.5	1578	27.5	108	33.8	
Body mass index (kg/m^2^)												
Mean (SD)		24.1	3.00	25.7	3.11	24.4	2.97	23.7	2.85	22.3	2.86	<1.0E-30
<23		4188	38.1	231	20.3	1371	34.1	2392	43.3	194	62.6	<1.0E-30
23–24.9		2927	26.6	261	23.0	1073	26.7	1529	27.7	64	20.6	
25+		3876	35.3	644	56.7	1582	39.3	1598	29.0	52	16.8	
Missing		487		70		190		217		10		
Weight (kg)												
Mean (SD)		61.6	8.10	64.8	8.15	62.2	8.06	60.7	7.86	56.7	7.46	<1.0E-30
Missing		281		55		109		111		6		
Height (cm)												
Mean (SD)		159.8	4.52	159.2	4.72	159.8	4.54	160.0	4.44	159.5	4.57	6.1E-09
Missing		229		19		89		116		5		
Age at menarche												
<13		1334	11.7	112	9.3	438	10.4	727	12.7	57	17.9	1.4E-20
13–14		5245	45.8	469	39.1	1862	44.3	2749	48.0	165	51.7	
15+		4874	42.6	620	51.6	1907	45.3	2250	39.3	97	30.4	
Missing		25		5		9		10		1		
Parity												
Never		683	6.0	37	3.1	229	5.4	380	6.6	37	11.6	3.0E-09
Ever		10795	94.1	1169	96.9	3987	94.6	5356	93.4	283	88.4	
Age at first full term birth^c^												
<25		3500	32.5	457	39.3	1276	32.1	1681	31.5	86	30.4	2.5E-04
25–26		3433	31.9	344	29.6	1277	32.1	1720	32.2	92	32.5	
27–28		2136	19.8	217	18.6	796	20.0	1068	20.0	55	19.4	
29+		1695	15.8	146	12.5	626	15.7	873	16.3	50	17.7	
Missing		31		5		12		14		0		
Breastfeeding^c^												
No breastfeeding		1870	17.3	174	14.9	645	16.2	982	18.3	69	24.4	2.5E-27
1–6 months		1866	17.3	156	13.3	699	17.5	952	17.8	59	20.8	
7–12 months		4276	39.6	396	33.9	1564	39.2	2200	41.1	116	41.0	
13 months+		2783	25.8	443	37.9	1079	27.1	1222	22.8	39	13.8	
Menopausal status												
Premenopausal		4100	35.7	154	12.8	1099	26.1	2665	46.5	182	56.9	<1.0E-30
Postmenopausal		7378	64.3	1052	87.2	3117	73.9	3071	53.5	138	43.1	
Age at menopause among postmenopausal women												
<49		2766	37.5	396	37.7	1134	36.4	1172	38.2	64	46.4	<1.0E-30
49–50		2613	35.4	347	33.0	1107	35.5	1116	36.4	43	31.2	
51+		1997	27.1	308	29.3	876	28.1	782	25.5	31	22.5	
Missing		2		1				1				
Smoking												
Never		10008	87.2	1075	89.1	3695	87.6	4957	86.4	281	87.8	0.01
Current		1264	11.0	105	8.7	435	10.3	689	12.0	35	10.9	
Former		206	1.8	26	2.2	86	2.0	90	1.6	4	1.3	
Alcohol consumption												
Never		8456	73.7	920	76.3	3140	74.5	4154	72.4	242	75.6	0.03
Current		2640	23.0	257	21.3	935	22.2	1383	24.1	65	20.3	
Former		381	3.3	28	2.3	141	3.3	199	3.5	13	4.1	
Missing		1		1								
Regular tea drinking												
No		6087	57.9	568	51.5	2185	56.4	3137	59.9	197	68.4	1.9E-09
Yes		4419	42.1	534	48.5	1692	43.6	2102	40.1	91	31.6	
Missing		972		104		339		497		32		

^a^Results from one-way analysis of variance (ANOVA) for continuous variables and *χ*^2^ test for categorical variables

^b^Excluding women with first-degree relatives with breast cancer <50 years

^c^Parous women only

Table 2 shows the overall associations between examined risk factors and MD in the multivariable polytomous regression model. Older age (OR_trend_ = 0.80, 95% CI = 0.79-0.82; per 2-year increase), postmenopausal status (OR_trend_ = 0.73, 95% CI = 0.66-0.81), higher BMI (OR_trend_ = 0.78, 95% CI = 0.76-0.80; per 2 kg/m^2^), having first-degree relatives with breast cancer diagnosed before age 50 (OR = 0.87, 95% CI = 0.80-0.96), and being parous (OR_trend_ = 0.74, 95% CI = 0.62-0.88) were associated with lower MD. In contrast, higher education level was associated with higher MD. Later first full term-birth was associated with higher MD (OR_trend_ = 1.38, 95% CI = 1.21–1.57, *P* = 1.2E-06; 29+ vs. <25 years). Women who had breastfed for longer than a year were likely to have lower MD (OR_trend_ = 0.84, 95% CI = 0.74-0.96). When weight and height were analyzed separately to replace BMI in the model, height was positively associated with MD (OR_trend_ = 1.10, 95% CI = 1.08-1.12; per 2 cm increase) and weight was inversely associated with MD (OR_trend_ = 0.91, 95% CI = 0.90-0.92; per 2 kg increase). Tea drinking was associated with lower MD in the multivariable model without the adjustment of province (OR_trend_ = 0.72, 95% CI = 0.67–0.78; yes vs. no), however, the association became non-significant (OR_trend_ = 0.98, 95% CI = 0.90-1.07) with the adjustment due to the significant heterogeneity by province.

**Table 2:** Associations between selected characteristics and mammographic density based on polytomous logistic regression in Chinese Breast Cancer Screening Program

	BI-RADS 2 vs. 1	BI-RADS 3 vs. 1	BI-RADS 4 vs. 1	OR_trend_ (95% CI)^b^	*P*_trend_^b^
	OR (95% CI)^a^	OR (95% CI)^a^	OR (95% CI)^a^		
Age					
Per 2 years	0.84 (0.82–0.87)	0.70 (0.68–0.72)	0.64 (0.60–0.68)	0.80 (0.79–0.82)	<1.0E-30
Family history of breast and ovarian cancers					
1st or 2nd degree relatives^c^	1.06 (0.87–1.29)	1.07 (0.87–1.32)	1.28 (0.89–1.84)	1.05 (0.94–1.16)	0.02
1st degree relatives with breast cancer <50 years	0.85 (0.71–1.00)	0.80 (0.67–0.96)	0.61 (0.43–0.87)	0.87 (0.80–0.96)	4.6E-04
Education					
Middle school vs. none-elementary	1.62 (1.33–1.98)	2.13 (1.71–2.67)	2.08 (1.18–3.66)	1.61 (1.41–1.83)	1.4E-12
High school vs. none-elementary	2.00 (1.62–2.47)	2.85 (2.26–3.59)	3.72 (2.15–6.42)	1.84 (1.61–2.10)	1.1E-19
College +vs. none-elementary	2.40 (1.86–3.11)	4.44 (3.36–5.86)	6.41 (3.59–11.45)	2.34 (2.03–2.71)	<1.0E-30
Body mass index					
Per 2 kg/m^2^	0.78 (0.74–0.81)	0.66 (0.63–0.69)	0.44 (0.40–0.49)	0.78 (0.76–0.80)	<1.0E-30
Height^d^					
Per 2 cm	1.13 (1.09–1.17)	1.19 (1.15–1.24)	1.33 (1.24–1.43)	1.10 (1.08–1.12)	1.1E-23
Weight^d^					
Per 2 kg	0.91 (0.89–0.93)	0.85 (0.83–0.87)	0.73 (0.70–0.76)	0.91 (0.90–0.92)	<1.0E-30
Age at menarche					
13-14 vs. <13	1.14 (0.88–1.48)	1.07 (0.82–1.40)	0.91 (0.60–1.38)	0.96 (0.85–1.10)	0.56
15 + vs. <13	1.07 (0.83–1.39)	0.95 (0.73–1.25)	0.74 (0.47–1.16)	0.90 (0.79–1.03)	0.13
Parity					
Parous vs. nulliparous	0.67 (0.46–0.98)	0.58 (0.39–0.86)	0.34 (0.20–0.59)	0.74 (0.62–0.88)	4.9E-04
Age at first full term birth^e^					
25–26 vs. <25	1.37 (1.14–1.66)	1.45(1.19–1.78)	1.47 (1.02–2.12)	1.17 (1.06–1.30)	2.2E-03
27–28 vs. <25	1.32 (1.06–1.64)	1.50 (1.19–1.89)	1.49 (0.96–2.30)	1.22 (1.08–1.37)	9.3E-04
29+ vs. <25	1.55 (1.21–1.99)	1.89 (1.46–2.45)	2.09 (1.31–3.33)	1.38 (1.21–1.57)	1.2E-06
Breastfeeding^e^					
1–6 months vs. no breastfeeding	1.42 (1.08–1.85)	1.40 (1.06–1.85)	1.26 (0.80–1.99)	1.07 (0.94–1.23)	0.31
7–12 months vs. no breastfeeding	1.30 (1.04–1.64)	1.38 (1.08–1.74)	1.21 (0.81–1.81)	1.12 (0.99–1.25)	0.07
13 months+ vs. no breastfeeding	0.99 (0.78–1.26)	0.87 (0.68–1.13)	0.45 (0.27–0.75)	0.84 (0.74–0.96)	0.01
Menopausal status					
Postmenopausal vs. Premenopausal	0.73 (0.58–0.92)	0.55 (0.44–0.70)	0.40 (0.28–0.59)	0.73 (0.66–0.81)	3.5E-09

^a^Polytomous logistic regression was used to estimate ORs and 95% CIs comparing each of the higher BI-RADS categories (2, 3, 4) to BI-RADS = 1, adjusted for province, age, family history, education, BMI, age at menarche, parity, and menopausal status

^b^Cumulative logistic regression (CLOGIT function) and Wald *χ*^2^ test were used to estimate OR_trend_ and *P*_trend_, with MD modeled as an ordinal variable comparing higher to lower BI-RADS categories (with BI-RADS = 1 as the reference). Model was adjusted for the same factors as above

^c^Excluding women with first-degree relatieves with breast cancer <50 years

^d^BMI was replaced with height and weight in the model

^e^Parous women only. Model was adjusted for province, age, family history, education, BMI, age at menarche, age at first full term birth, breastfeeding, and menopausal status

The distributions of risk factors for dense and non-dense groups are shown in Supplementary Table 1. Overall, associations for most risk factors examined were similar in premenopausal and postmenopausal women except for family history, parity and breastfeeding duration (Table 3). The association of family history (first-degree relatives with breast cancer before age 50) was associated with MD only among premenopausal women (OR = 0.75, 95% CI = 0.62–0.91) but not among postmenopausal women (OR = 1.01, 95% CI = 0.89–1.15). In contrast, statistically significant associations with parity and breastfeeding were only observed among postmenopausal women with the interaction term significant only for breastfeeding. In addition, among postmenopausal women, later age at menopause was associated with higher MD (OR_trend_ = 1.12, 95% CI = 1.05–1.20).

**Table 3:** Associations between selected characteristics and mammographic density (BI-RADS 3–4 versus BI-RADS 1–2) by menopausal status

	Premenopausal (*n* = 4100)		Postmenopausal (*n* = 7378)	
	OR (95% CI)^a,b^	*P*	OR (95% CI)^a,c^	*P*
Age				
Per 2 years	0.81 (0.78–0.84)	3.1E-27	0.80 (0.78–0.82)	<1.0E-30
Family history of breast and ovarian cancers				
1st or 2nd degree relatives^d^	0.94 (0.76–1.17)	0.42	1.07 (0.93–1.24)	0.34
1st degree relatives with breast cancer <50 years	0.75 (0.62–0.91)	0.01	1.01 (0.89–1.15)	0.72
Education				
Middle school vs. none-elementary	1.25 (0.90–1.72)	0.18	1.51 (1.26–1.80)	5.7E-06
High school vs. none-elementary	1.27 (0.92–1.76)	0.14	1.76 (1.47–2.10)	4.6E-10
College +vs. none-elementary	1.73 (1.24–2.41)	0.001	2.29 (1.88–2.80)	3.0E-16
OR_trend_ (95 CI)^1^; *P*_trend_	1.18 (1.08–1.29)	0.000	1.28 (1.21–1.36)	2.5E-16
Body mass index				
Per 2 kg/m^2^	0.78 (0.74–0.82)	1.2E-19	0.82 (0.79–0.85)	2.1E-26
Height^e^				
Per 2 cm	1.09 (1.05–1.14)	4.7E-06	1.07 (1.05–1.10)	1.2E-07
Weight^e^				
Per 2 kg	0.91 (0.89–0.93)	2.4E-19	0.92 (0.91–0.94)	7.9E-26
Age at menarche				
13–14 vs. <13	1.01 (0.80–1.26)	0.97	0.90 (0.74–1.08)	0.26
15+ vs. <13	0.85 (0.67–1.08)	0.18	0.88 (0.73–1.07)	0.20
OR_trend_ (95 CI)^a^; *P*_trend_	0.90 (0.81–1.01)	0.08	0.95 (0.88–1.04)	0.28
Parity				
Ever vs. never	0.87 (0.62–1.21)	0.40	0.77 (0.61–0.97)	0.02
Age at first full term birth^e,f^				
25–26 vs. <25	1.04 (0.86–1.26)	0.69	1.13 (0.98–1.30)	0.09
27–28 vs. <25	1.09 (0.86–1.38)	0.48	1.21 (1.03–1.42)	0.02
29+ vs. <25	1.29 (1.00–1.66)	0.05	1.29 (1.08–1.54)	0.01
OR_trend_ (95 CI)^a^; *P*_trend_	1.08 (1.00–1.17)	0.06	1.09 (1.03–1.15)	2.6E-03
Breastfeeding^e,f^				
1-6 months vs. no breastfeeding	0.86 (0.67–1.11)	0.24	1.13 (0.93–1.36)	0.21
7-12 months vs. no breastfeeding	1.28 (1.03–1.60)	0.03	0.98 (0.84–1.16)	0.85
13 months +vs. no breastfeeding	0.98 (0.75–1.26)	0.86	0.79 (0.66–0.95)	0.01
OR_trend_ (95 CI)^a^; *P*_trend_	1.05 (0.97–1.14)	0.26	0.92 (0.87–0.97)	4.0E-03
Age at menopause among postmenopausal women				
49–50 vs. <49	–	–	1.12 (0.99–1.27)	0.94
51+ vs. <49	–	–	1.26 (1.10–1.44)	5.2E-03
OR_trend_ (95 CI)^a^; *P*_trend_	–	–	1.12 (1.05–1.20)	1.1E-03

^a^Logistic regression was used to estimate ORs and 95% CIs comparing the dense (BI-RADS = 3, 4) to non-dense breast group (BI-RADS = 1, 2). OR_trend_ and *P*_trend_ were obtained by treating categorical variables as ordinal variables

^b^Adjusted for province, age, family history, education, BMI, age at menarche, and parity

^c^Adjusted for province, age, education, BMI, age at menarche, parity, and age at menopause

^d^Excluding women with first-degree relatives with breast cancer <50 years

^e^BMI was replaced with height and weight in the model

^f^Parous women only. Adjusted for province, age, menopausal status, education, BMI, age at menarche, age at first full term birth, and breastfeeding

**Fig. 2:**
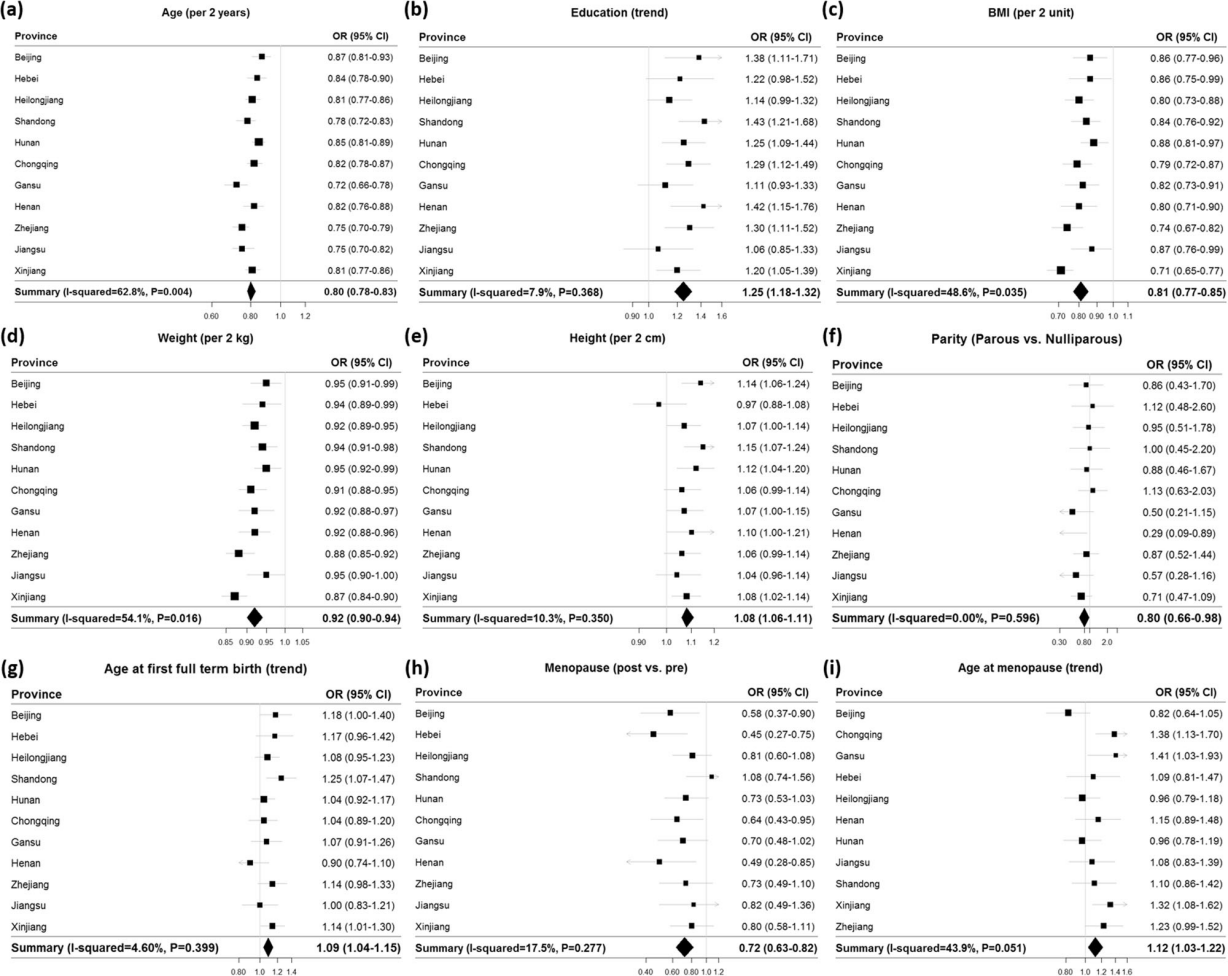
Associations of selected risk factors with mammographic density by province: **a** age, **b** education, **c** BMI, **d** weight, **e** height, **f** parity, **g** age at first full-term birth, **h** menopausal status, and **i** age at menopause. Province-specific OR_trend_ and *P*_trend_ for having dense (BI-RADS, 3–4) compared to non-dense breast (BI-RADS, 1–2) were estimated using multivariable logistic regression adjusted for age (per 2-years), education (none to elementary, middle school, high school, college + ), BMI (per 2 kg/m^2^), age at menarche (<13, 13-14, 15+years old), parity (parous vs. nulliparous), and menopausal status (premenopausal and postmenopausal). Age at full-term birth (<25, 25–26, 27–28, 29+) was estimated only for parous women with additional adjustment for breastfeeding duration (no breastfeeding, 1–6 months, 7–12 months, 13 months+). OR for age at menopause (<49, 49-50, 51+) was estimated only for postmenopausal women. For categorical variables (education, age at first full term birth, and age at menopause), linear trend in association (*P*_trend_) was tested by treating each factor as an ordinal variable. Meta-analysis was performed based on the random-effects model

**Fig. 3:**
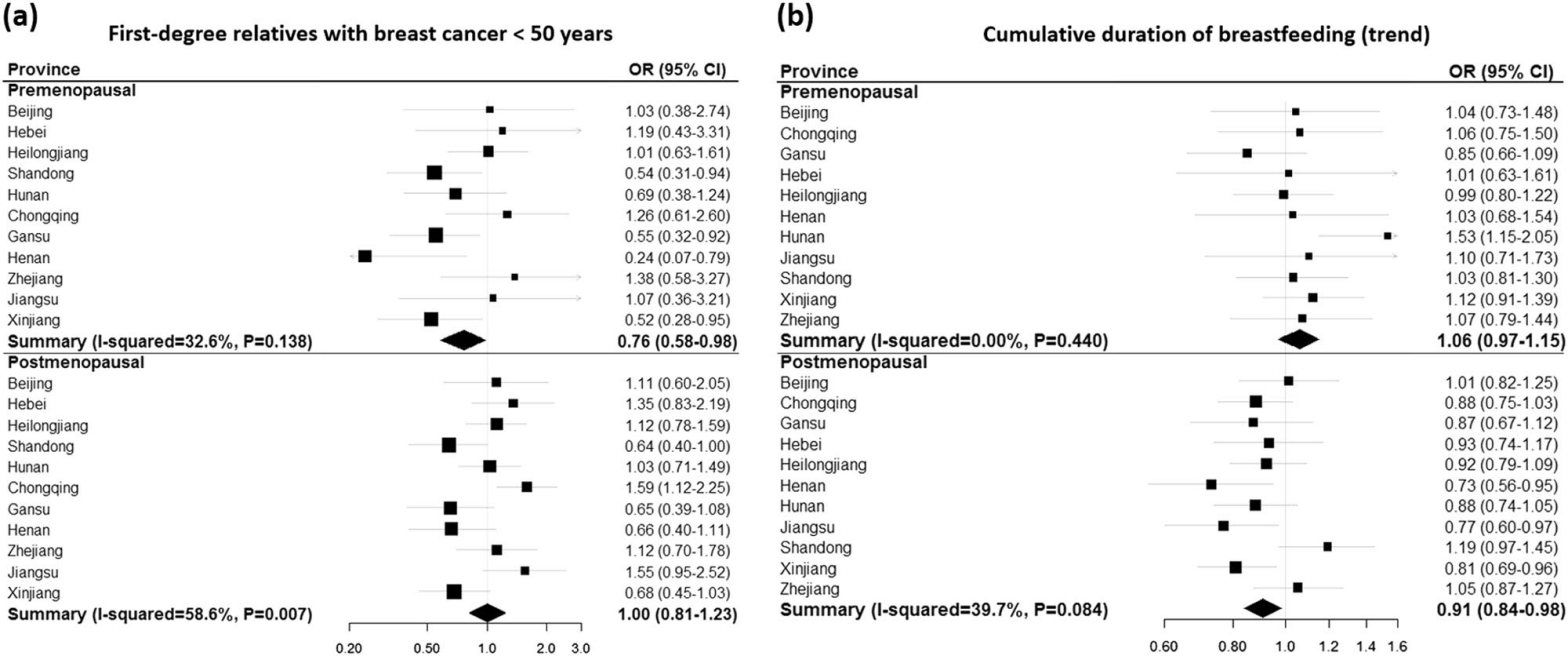
Associations of **a** having a first-degree relative with breast cancer before age 50 and **b** breastfeeding duration with mammographic density by province and menopausal status. Province-specific OR (95% CI) for having dense breast (BI-RADS, 3–4) compared with non-dense breast (BI-RADS, 1–2) was estimated by multivariable logistic regression. Meta-analysis was performed based on the random-effect model. **a** Group without family history of either breast or ovarian cancer was used as the reference. Model was adjusted for age (per 2 years), education (none to elementary, middle school, high school, college+), BMI (per 2 kg/m^2^), age at menarche (<13, 13-14, 15+years old), and parity (parous vs. nulliparous). Model for postmenopausal women was additionally adjusted for age at menopause (<49, 49-50, 51+). **b** Breastfeeding duration (no breastfeeding, 1–6 months, 7–12 months, 13 months+) was treated as an ordinal variable among parous women with the adjustment for age (per 2 years), family history (no history, first- and/or second-degree relatives with breast and/or ovarian cancers except for first-degree with breast cancer before age 50 years, and first-degree relative with breast cancer before age 50 years; premenopausal women only), education (none to elementary, middle school, high school, college+), BMI (per 2 kg/m^2^), age at menarche (<13, 13–14, 15+years old), age at full-term birth (<25, 25–26, 27–28, 29+), and age at menopause (<49, 49–50, 51+; postmenopausal women only)

Figure 2 shows province-specific associations from the meta-analysis. Among all risk factors examined, the associations for MD with age, BMI and weight differed in magnitude, but not direction by province (*P*_heterogeneity_ < 0.05). Figure 3 shows province-specific associations for family history and breastfeeding and MD by menopausal status. The associations between MD and having a first-degree relative with breast cancer before age 50 showed heterogeneity by province (Fig. 3a, *P*_heterogeneity_ = 0.14 and 0.007 for premenopausal and postmenopausal women, respectively), whereas the associations with breastfeeding did not show significant variation across provinces in either premenopausal or postmenopausal women (Fig. 3b).

## Discussion

The goal of this study was to examine associations of MD with known breast cancer risk factors using data collected from a population-based multi-center screening program in China. Our study is one of the largest MD evaluations conducted among Asian women, which permitted stratification by menopausal status. Our results are consistent with the only other large study conducted among 28,388 Chinese, showing that MD was related to age, menopause, parity, and age at first full-term birth.^41^ However, the previous study did not collect information on several important risk factors (such as breastfeeding and age at menopause, etc.) and did not investigate the potential modification by menopausal status. Using a more comprehensive multivariable model, in addition to those expected associations, we observed that increasing height, higher education level, and later age at menopause were associated with higher MD, whereas having first-degree relatives with breast cancer diagnosed before age 50 was associated with lower MD. Further, we found that longer duration of breastfeeding was associated with lower MD but only among postmenopausal women. Taken together, these findings support the hypothesis that some established breast cancer risk factors may be mediated through MD, an intermediate phenotype for breast cancer risk, among Chinese women.

Similar associations of MD with age, weight, BMI, and parity observed in Asian and Western countries suggest that these factors have a similar impact on MD in different populations.^29–42,45,46^ However, it remains unclear whether the effect size for these MD-influencing factors differs by race/ethnicity. A longitudinal analysis showed that the age-related decline of PMD was slower among Japanese women compared to Caucasians,^47^ suggesting possible racial/ethnic heterogeneity in MD variations in relation to age. Similarly, although some researchers observed a strong inverse correlation between BMI (or weight) and PMD across diverse populations,^22,40,48–50^ the associations between absolute areas (dense or non-dense) and BMI varied significantly by ethnicity,^22,30,32,40,50,51^ which may be due to the substantial difference in body size and breast composition by ethnicity.^29^ Standardized quantitative MD measurements in diverse populations are needed to address this question.

The inverse associations of age and BMI with MD have been consistently found and was also confirmed in our study. The seemingly paradoxical age association was explained previously by the concept of ‘breast tissue aging (exposure)’ proposed by Pike.^46^ Specifically, in Pike’s model, the decline of PMD with age parallels that of the rate of ‘breast tissue aging’, which refers to the cumulative exposure of breast tissue to hormones, growth factors, and other carcinogens and was suggested as a more relevant measure in relation to MD compared with chronological age.^45,46^ In this model, both the rates of breast tissue aging and PMD are greatest at younger ages and decline with increasing (chronological) age, and both show decreases during pregnancy and menopause. The inverse association between BMI and MD likely reflects the correlation between body fat and fatty tissue within breasts. Previous studies have shown that the effects of weight and MD on breast cancer risk both became stronger after the adjustment for each other, suggesting that BMI and MD are negative confounders of each other in their associations with the risk of breast cancer and likely to operate via independent pathways.^50^

A recent meta-analysis estimated that breast cancer risk increased by 17% with every 10 cm increase in height^52^ and endogenous factor such as IGF-1 have been linked to underlying height, MD and breast cancer risk.^53,54^ However, prior evaluations of MD and height have led conflicting results; positive^22,55^ and null associations^48,50^ have been reported in both Asian and Western populations. More studies are needed to confirm the height–MD association.

The genetic basis of MD remains largely unknown. Previous studies showed that women with higher MD were more likely to have first-degree relatives with breast cancer^56^ and odds increased with the number of affected relatives.^57,58^ A recent genome-wide association study evaluating common genetic variants also identified shared genetic loci associated with both breast cancer risk and MD.^59^ Therefore, the unexpected association of lower density with positive family history of breast cancer observed in our study requires cautious interpretation. It is noteworthy that a recent Spanish study showed MD was significantly lower among *BRCA2* mutation carriers compared to non-carriers in women from high-risk families.^60^ Therefore, it is possible that high-risk rare mutations and low-risk common variants may have different effect on MD. This notion is consistent with our observation of the significant association only among premenopausal women and women with first-degree earlier-onset family history, i.e., women more likely to carry high-risk alleles. In addition, our study participants were selected to be enriched for positive family history of breast and/or ovarian cancers, and therefore, the MD-family history association found in our study may not generalize to the general population.

Consistent with several studies,^61,62^ we found that higher levels of education were independently associated with higher MD, suggesting that the MD association with education could not be fully accounted for by measured variables such as BMI and reproductive factors. Other unmeasured lifestyle factors including dietary factors, physical activity, and socioeconomic status-related stress may also contribute and further investigation of these relationships is warranted in future studies.

We found that menopausal status was independently associated with MD after accounting for age and other confounders. This finding is consistent with previous evidence showing that age-related declines in MD are non-linear and that the rate of change may be faster among younger than older women.^63^ In our study, we also found that earlier age at menarche and later age at menopause were associated with higher MD, which supports the hypothesis that the cumulative exposure to endogenous hormones has a similar impact on MD and breast cancer risk.^46^ Likewise, the associations of nulliparity and older age at first full-term birth with higher MD also suggest that hormone-related risk factors may potentially affect breast cancer risk through MD.

The literature regarding breastfeeding and MD is inconsistent,^32,35,38,42,64,65^ which may be due to limited sample sizes in these studies, as well as a lack of information on breastfeeding duration. We observed that longer breastfeeding was associated with lower MD among postmenopausal women. The reason for difference by menopausal status is not clear, but it may partly involve the long-term reduction in prolactin levels among parous women,^66^ as circulating prolactin levels have been positively associated with MD among postmenopausal women in other studies.^67,68^ On the other hand, the lack of significant association among premenopausal women may be due to the relatively lower proportion of women with longer breastfeeding duration (≥1years, 21.1% among premenopausal vs. 28.4% among postmenopausal women). Additionally, we cannot exclude the possibility that the association with breastfeeding is mediated through parity since older Chinese women would have been more likely to have more children than younger women because of the one-child policy in China that started in the late 1970’s. This is an area for future exploration.

Previous studies reported associations of MD with lifestyle factors such as alcohol consumption,^69,70^ smoking,^19,69^ and tea,^71^ however, these factors were not associated with MD in our study. We found that tea drinkers were less likely to have dense breast, which is a potentially interesting finding since tea drinking has been shown to be protective for breast cancer by previous studies and the prevalence of tea drinking is high in China. However, the association was only seen in a few provinces (Chongqing and Gansu) and there was significant heterogeneity across provinces resulting in an overall lack of association after adjusting for province. In addition, detailed tea drinking behaviors (frequency, amount, duration, etc.) were not collected in this study. Future studies with detailed exposure measures are warranted to clarify the relationship between tea consumption and MD in Asian populations.

Major strengths of our study include the large number of study participants in an understudied Asian population, a comprehensive collection and analysis of multiple exposures of interest, centralized data collection, management, quality control, and quality assurance. All participating sites are first class tertiary hospitals in each province and all radiologists participating in the screening program have radiology certificate from the Department of Health and are required to have at least 5 years of clinical experiences in breast imaging. In addition, all participating radiologists received an intensive training before the screening program started followed with on-site training sessions during the screening program to enforce uniform standards for mammographic procedures and diagnosis and density reporting. Additionally, multi-regional participation has provided a distinct opportunity to assess regional heterogeneity in MD influencing factors. Several limitations of our study should be noted. First, MD was assessed using BI-RADS, which is subjective and known to vary across radiologists.^72,73^ Despite the centralized and on-site training to enforce the uniform MD measurement, measurement variability across different radiologists and screening sites is likely to exist. This subjectivity and variability may cause potential misclassification of MD levels and confound MD-risk factor associations. In addition, the MD associations observed in our study may also be confounded by other unmeasured or unknown factors such as lifestyle, social, and environmental factors and these factors are likely to vary across provinces (urbanization, air pollution, occupational exposure, etc.), which may partly explain the observed heterogeneity by province. Nevertheless, the associations between most factors examined and MD were consistent across different regions, suggesting that the observed associations were internally valid. Our study was also limited by the categorical BI-RADS measurement and the two-dimensional view. A quantitative measurement that assesses different phenotypes of MD (e.g., PMD, absolute dense area, non-dense area) accompanied by three-dimensional views or a volumetric approach may be more useful in establishing relationships between breast cancer risk factors and MD. Additionally, mammography was only performed on urban Chinese women who were predicted at being high-risk for breast cancer, and thus they are not representative of the general population. This design may account for the slightly higher proportion of dense breasts (BI-RADS 3 and 4; 54%) compared with the previous report (49.2%) of Chinese women within the same age groups.^41^

In summary, we found that later age at first full-term birth, later age at menopause, height, and higher education level were positively associated with MD, and age, BMI, family history of breast cancer, parity, and longer durations of breastfeeding were negatively related to MD among Chinese women at elevated breast cancer risk. Except for age, BMI, and family history of breast cancer, other established breast cancer risk factors showed the same direction of effect on MD, suggesting that the effect of these risk factors may be mediated through their influence on MD, an intermediate phenotype for breast cancer. Most associations were similar for premenopausal and postmenopausal women, except for breastfeeding duration, which showed stronger association among postmenopausal women. Further studies using standardized MD measures in diverse populations are warranted and may provide useful information to identify high-risk women particularly in low-resource countries where mammographic screening is not readily available to the general population.

## Methods

### Study population

**Fig. 4:**
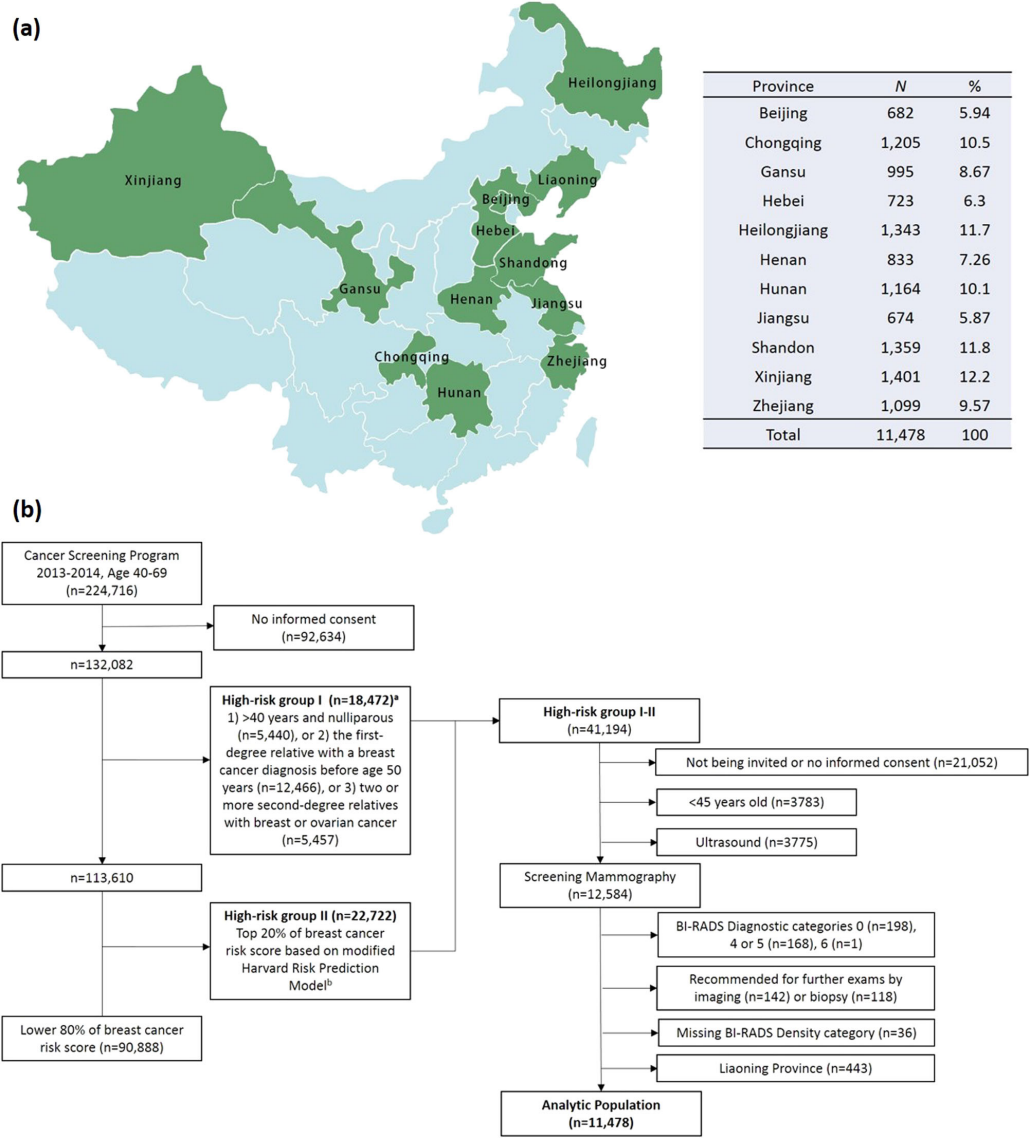
Schematic of the process of selecting high-risk women from the Chinese Breast Cancer Screening Program (2013-2014) for mammogram screening and inclusion in the density evaluation: **a** 12 participating provinces and number of participants by province **b** flowchart, ^a^Not mutually exclusive, ^b^Risk factors included in the prediction model were: body mass index (BMI), long-term mental depression, age at menarche, menstrual irregularity, age at menopause, age at first marriage, breastfeeding duration, history of benign breast disease, previous surgeries of the reproductive system, and family history of breast cancer. Note: Liaoning (*n* = 443) was not included in the final analysis

Study subjects were a subset of participants aged 40–69 in an ongoing cancer screening program in urban China led by the National Cancer Center/Cancer Hospital, Chinese Academy of Medical Sciences (CHCAMS). The goal of this program is to screen for the five most common cancers in China, including lung, breast, colorectal, upper digestive tract, and liver cancers. The program was initiated in 2012 and covered 12 provinces (Fig. 4a) in China between 2013 and 2014. Participants were interviewed to obtain risk factor data for the five targeted cancers using a standardized questionnaire. A risk prediction strategy was used to estimate risk for each cancer site. Individuals considered to be at high-risk for one of these five cancers were invited for further screening.

For breast cancer, two sequential methods were used to define high-risk groups. The first used information on family history of breast or ovarian cancer and parity, and the second using risk prediction software (Remote Data Collection System and Cancer Risk Assessment System) modified from the Harvard Risk Prediction Model^74^ based on prevalence of risk factors and relative risks in the Chinese population (Fig. 4b). A subset of women determined to be at high-risk for breast cancer were invited, on a first-come first-served basis, to participate in the breast cancer screening by ultrasound and/or mammographic imaging (only for women ≥ 45 years).

This study included women who participated in the screening program in 2013–2014 and had mammogram data available (Fig. 4b). Our eligible sample included 12,584 women from 17 cities located in one of 12 provinces (or municipalities): Beijing, Chongqing, Gansu, Hebei, Heilongjiang, Henan, Hunan, Jiangsu, Liaoning, Shandong, Xinjiang, and Zhejiang. We restricted study subjects to women with negative imaging findings (i.e., Breast Imaging Reporting and Data System (BI-RADS) diagnostic categories, 1, 2 or 3). We excluded women if they had incomplete diagnostic classification (BI-RADS diagnostic category = 0) or suspected/confirmed malignant tumors (BI-RADS diagnostic category = 4, 5, 6), were recommended for further exams by imaging, or had missing density data. We additionally excluded participants from Liaoning province (*n* = 443) since most women (96%) were classified as having heterogeneously dense breasts (BI-RADS density score = 3) and we suspected potential MD misclassification. Our final analytic sample included a total of 11,478 women. The distribution of number of participant, age, and menopausal status by province was shown in Supplementary Table 2. The mean age ranged from 52.8 in Xinjiang to 55.5 in Chongqing, and the proportion of postmenopausal women varied from 58% in Xinjiang to 71% in Chongqing. The study was approved by the Institutional Review Board of CHCAMS and informed consent was obtained from all participants in the study. The research was conducted following all relevant guidelines and procedures. This study was exempted from review by the Office of Human Subject Research Protections at the National Institutes of Health since NIH investigators do not have the access to the personal identifying information (Exempt Number: 13328).

### Mammographic density assessment

Full-field digital mammograms were obtained in each participating hospital. A total of four films capturing craniocaudal and mediolateral oblique views of both breasts were obtained for each woman. Imaging results were read by trained radiologists in each designated hospital and categorized using the BI-RADS guidelines (BI-RADS diagnostic category (0–6) and BI-RADS density scoring system (1–4)) recommended by the American College of Radiology (4th edition).^75^ MD was categorized into four levels using the BI-RADS density scoring system: almost entirely fat (1), scattered fibroglandular densities (2), heterogeneously dense (3), and extremely dense (4). Maximal values for BI-RADS diagnostic and density categories were used across the two breasts.

### Selected risk factors

Data on breast cancer risk factors were extracted from the baseline questionnaire, including age, family history of breast and/or ovarian cancers, education level, lifestyle factors (smoking, alcohol consumption, and regular tea drinking), and reproductive history (age at menarche, parity, age at first full-term birth, cumulative months of breastfeeding for all live-born children, menopausal status, and age at menopause). Height (cm) and weight (kg) were measured and BMI was calculated from weight and height as kg/m^2^.

### Statistical analysis

Differences in the distribution of each risk factor by BI-RADS density categories were assessed using one-way analysis of variance (ANOVA) or *χ*^2^ test. To assess associations between MD and risk factors, we used polytomous logistic regression to compare each higher MD category (BI-RADS 2, 3, and 4) to BI-RADS = 1 (almost entirely fat, the referent group). Odds ratios (ORs) and corresponding 95% confidence intervals (CIs) were estimated using the PROC LOGISTIC procedure with the GLOGIT option. OR_trend_ (95% CI, *P*_trend_) across BI-RADS categories were also obtained using cumulative logistic regression with treating BI-RADS categories as an ordinal variable.

The base model included age, BMI, and province as covariates. For the multivariable analysis, we included all variables that had *P* < 0.05 in the base model. The final multivariable model included province, age (per 2 years), family history of breast and/or ovarian cancers (no history, first-degree relative with breast cancer before age 50 years, and other history), menopausal status (pre- or postmenopausal), education (none to elementary, middle school, high school, or college+), BMI (per 2 kg/m^2^), age at menarche ( < 13, 13–14, or 15+ years old), and parity (parous or nulliparous). Among parous women, age at first full-term birth (<25, 25–26, 27–28, or 29+ years), and breastfeeding duration (no breastfeeding, 1–6 months, 7–12 months, or 13 months+) were also included in the model. We also ran models in which we replaced BMI with height (per 2 cm) or weight (per 2 kg) to examine height and weight separately. Variables such as smoking (never, former, or current), alcohol consumption (never, former, or current), and regular tea drinking (yes or no) were not associated with MD in base model and therefore, were excluded from the final multivariable model. In the sensitivity analysis, the additional adjustment for these factors did not change the magnitude of the associations with other variables (data not shown).

Because density is known to decrease with menopause,^43^ we therefore stratified our analysis by menopausal status. In stratified analyses, BI-RADS categories were collapsed into dense (BI-RADS, 3 and 4) and non-dense (BI-RADS, 1 and 2, reference) groups because of small number in some extreme categories. Models for postmenopausal women were additionally adjusted for age at menopause (<49, 49-50, or 51+). The potential effect modification by menopausal status was tested by including an interaction term between each explanatory variable and menopausal status in the analysis. Since family history was associated with MD only among premenopausal women, it was only included in the analysis for premenopausal women.

To assess the potential heterogeneity across different provinces, we also stratified analyses by province and provided summary statistics using meta-analysis based on a random-effects model. Tests for heterogeneity across provinces were assessed by *I*^2^ statistics.

All statistical tests were two-sided. Meta-analysis was conducted using Stata/SE (version 11.2; StataCorp LP, College Station, TX) and all other analyses were conducted using SAS (version 9.3; SAS Institute Inc., Cary, NC).

### Data and code availability statement

The data sets and code used and analyzed for the current study will be available from the corresponding author upon reasonable request without undue qualifications.

## Electronic supplementary material

Supplementary Material Legend

Supplementary Table 1

Supplementary Table 2

Supplementary Table 3
