# Supplementary material for: Breast cancer risk factors and mammographic density among high-risk women in urban China
Source: NPJ Breast Cancer. 2018 Feb 6;4:3. doi: 10.1038/s41523-018-0055-9 (PMC5802809; doi:10.1038/s41523-018-0055-9)
Supplement: Supplementary file 1 — Supplementary Material Legend [file 41523_2018_55_MOESM1_ESM.docx]

**Supplementary Materials**

**Supplementary Table 1.** Demographic characteristics, anthropometric measures, reproductive and lifestyle factors among participants of the Chinese breast cancer screening program by menopausal status

**Supplementary Table 2.** Distribution of age and menopausal status by province

**Supplementary Table 3.** Associations between selected characteristics and mammographic density (BI-RADS 3-4 versus BI-RADS 1-2)
