# Supplementary material for: Breast cancer risk factors and mammographic density among high-risk women in urban China
Source: NPJ Breast Cancer. 2018 Feb 6;4:3. doi: 10.1038/s41523-018-0055-9 (PMC5802809; doi:10.1038/s41523-018-0055-9)
Supplement: Supplementary file 2 — Supplementary Table 1 [file 41523_2018_55_MOESM2_ESM.pdf]

Supplementary Table 1. Demographic characteristics, anthropometric measures, reproductive and lifestyle factors among participants of the Chinese breast cancer screening program by menopausal status

	Premenopausal					Postmenopausal				
	BI-RADS 1-2 (n=1253)		BI-RADS 3-4 (n=2847)			BI-RADS 1-2 (n=4169)		BI-RADS 3-4 (n=3209)		
	N	%	N	%	*P*[a]	N	%	N	%	*P*[a]
**Age**										
Mean (SD)	50.2	4.69	48.6	3.30	<1.0E-30	58.5	5.37	55.7	5.24	<1.0E-30
45-49	654	52.2	1883	66.1	1.8E-25	159	3.8	320	10.0	<1.0E-30
50-59	520	41.5	920	32.3		2205	52.9	2115	65.9	
60-69	79	6.3	44	1.6		1805	43.3	774	24.1	
**Family history of breast and/or ovarian cancers**										
No	645	51.5	1582	55.6	0.009	2045	49.1	1525	47.5	0.41
1st or 2nd degree relatives[b]	228	18.2	528	18.6		812	19.5	637	19.9	
1st degree relatives with	380	30.3	734	25.8		1311	31.5	1047	32.6	
Missing			3			1				
**Education**										
None-Elementary	108	8.6	158	5.6	6.2E-09	781	18.7	322	10.0	<1.0E-30
Middle school	380	30.3	748	26.3		1343	32.2	934	29.1	
High school	460	36.7	995	35.0		1386	33.3	1213	37.8	
College+	305	24.3	946	33.2		659	15.8	740	23.1	
**Body mass index (kg/m^2)**										
Mean (SD)	24.6	3.06	23.5	2.82	6.2E-25	24.7	3.05	23.7	2.90	<1.0E-30
<23	380	32.1	1246	45.3	1.2E-20	1222	30.7	1340	43.5	<1.0E-30
23-24.9	308	26.0	755	27.5		1026	25.8	838	27.2	
25+	496	41.9	749	27.2		1730	43.5	901	29.3	
Missing	69		97			191		130		
**Weight (kg)**										
Mean (SD)	63.2	8.15	60.6	7.83	3.0E-21	62.7	8.15	60.3	7.94	<1.0E-30
Missing	50		49			114		68		
**Height (cm)**										
Mean (SD)	160.3	4.54	160.3	4.34	0.93	159.5	4.59	159.7	4.52	0.02
Missing										
**Age at menarche**										
<13	185	14.8	430	15.1	0.003	365	8.8	354	11.1	1.3E-08
13-14	551	44.1	1397	49.2		1780	42.8	1517	47.4	
15+	514	41.1	1014	35.7		2013	48.4	1333	41.6	
Missing	3		6			11		5		
**Parity**										
Never	67	5.4	193	6.8	0.08	199	4.8	224	7.0	5.3E-05
Ever	1186	94.7	2654	93.2		3970	95.2	2985	93.0	
**Age at first full term birth[c]**										
<25	436	36.9	866	32.7	0.03	1297	32.8	901	30.3	0.12
25-26	392	33.1	879	33.2		1229	31.1	933	31.3	
27-28	192	16.2	468	17.7		821	20.8	655	22.0	
29+	163	13.8	433	16.4		609	15.4	490	16.5	
Missing	3		8			14		6		
**Breastfeeding[c]**										
No breastfeeding	222	18.7	509	19.2	0.02	597	15.0	542	18.2	2.1E-12
1-6 months	253	21.3	474	17.9		602	15.2	537	18.0	
7-12 months	449	37.9	1122	42.3		1511	38.1	1194	40.0	
13 months+	262	22.1	549	20.7		1260	31.7	712	23.9	
**Age at menopause among postmenopausal women**										
<49	-	-	-	-	-	1530	36.7	1236	38.5	<1.0E-30
49-50	-	-	-	-		1454	34.9	1159	36.1	
51+	-	-	-	-		1184	28.4	813	25.3	
Missing						1		1		
**Smoking**										
Never	1103	88.0	2471	86.8	0.23	3667	88.0	2767	86.2	0.01
Current	131	10.5	343	12.1		409	9.8	381	11.9	
Former	19	1.5	33	1.2		93	2.2	61	1.9	
**Alcohol consumption**										
Never	889	71.0	2047	71.9	0.51	3171	76.1	2349	73.2	0.01
Current	311	24.8	700	24.6		881	21.1	748	23.3	
Former	53	4.2	100	3.5		116	2.8	112	3.5	
Missing						1				
**Regular tea drinking**										
No	599	51.7	1547	59.5	8.3E-06	2154	56.4	1787	61.0	1.2E-04
Yes	559	48.3	1052	40.5		1667	43.6	1141	39.0	
Missing	95		248			348		281		

[a]Results from one-way analysis of variance (ANOVA) for continuous variables and chi-squared test for categorical variables
[b]Excluding women with first-degree relatieves with breast cancer < 50 years
[c]Parous women only
