# Supplementary material for: Breast cancer risk factors and mammographic density among high-risk women in urban China
Source: NPJ Breast Cancer. 2018 Feb 6;4:3. doi: 10.1038/s41523-018-0055-9 (PMC5802809; doi:10.1038/s41523-018-0055-9)
Supplement: Supplementary file 3 — Supplementary Table 2 [file 41523_2018_55_MOESM3_ESM.pdf]

Supplementary Table 2. Distribution of age and menopausal status by provinces

Provinces	N	%	Age		Menopausal status (%)		BI-RADS Score (%)			
			Mean	SD	Premenopause	Postmenopause	1	2	3	4
Beijing	682	5.94	55.3	6.25	29.3	70.7	7.8	44.9	44.3	3.1
Chongqi	1205	10.5	55.5	6.51	29.0	71.0	7.3	27.7	56.7	8.3
Gansu	995	8.67	53.1	5.99	38.1	61.9	18.5	50.2	30.1	1.3
Hebei	723	6.3	54.1	6.41	39.8	60.2	3.5	24.3	71.7	0.6
Heilong	1343	11.7	53.8	5.93	42.1	57.9	4.6	41.7	53.4	0.3
Henan	833	7.26	54.1	6.38	37.7	62.3	3.1	20.5	68.4	7.9
Hunan	1164	10.1	55.1	6.60	32.7	67.4	6.7	38.3	52.1	2.9
Jiangsu	674	5.87	54.6	6.13	31.5	68.6	1.3	37.7	57.3	3.7
Shandon	1359	11.8	55.4	6.07	30.1	69.9	34.4	44.2	20.5	0.9
Xinjian	1401	12.2	52.8	6.23	41.9	58.1	8.4	37.3	53.0	1.3
Zhejian	1099	9.57	54.6	6.46	37.9	62.1	8.7	31.6	57.6	2.1
Total	11478	100	54.4	6.30	35.7	64.3	10.5	36.7	50.0	2.8
