# Supplementary material for: Breast cancer risk factors and mammographic density among high-risk women in urban China
Source: NPJ Breast Cancer. 2018 Feb 6;4:3. doi: 10.1038/s41523-018-0055-9 (PMC5802809; doi:10.1038/s41523-018-0055-9)
Supplement: Supplementary file 4 — Supplementary Table 3 [file 41523_2018_55_MOESM4_ESM.pdf]

Supplementary Table 3. Associations between selected characteristics and mammographic density (BI-RADS 3-4 versus BI-RADS 1-2)

	OR (95% CI)[a]	*P*
Age		
Per 2 years	0.81 (0.79- 0.82)	<1.0E-30
Family history of breast and ovarian cancers		
1st or 2nd degree relatives[b]	1.03 (0.91- 1.16)	0.65
1st degree relatives with breast cancer < 50 years	0.91 (0.82- 1.02)	0.09
Education		
Middle school vs. None-Elementary	1.46 (1.25- 1.70)	1.40E-06
High school vs. None-Elementary	1.64 (1.40- 1.91)	3.30E-10
College+ vs. None-Elementary	2.17 (1.83- 2.56)	1.50E-19
Body mass index (kg/m^2)		
Per 2kg/m^2	0.81 (0.78- 0.83)	<1.0E-30
Height (cm)[c]		
Per 2 cm	1.08 (1.06- 1.10)	1.90E-12
Weight (kg)[c]		
Per 2 kg	0.92 (0.91- 0.93)	<1.0E-30
Age at menarche		
13-14 vs. <13	0.95 (0.82- 1.09)	0.47
15+ vs. <13	0.89 (0.77- 1.03)	0.13
Parity		
Parous vs. Nulliparous	0.81 (0.67- 0.98)	0.03
Age at first full term birth[d]		
25-26 vs. <25	1.11 (0.99- 1.24)	0.07
27-28 vs. <25	1.19 (1.04- 1.35)	0.01
29+ vs. <25	1.30 (1.13- 1.50)	3.60E-04
Breastfeeding[d]		
1-6 months vs. No breastfeeding	1.02 (0.88- 1.19)	0.78
7-12 months vs. No breastfeeding	1.08 (0.95- 1.23)	0.22
13 months+ vs. No breastfeeding	0.86 (0.74- 0.99)	0.04
Menopausal status		
Post- vs. Premenopausal	0.73 (0.65- 0.82)	4.10E-08

[a]Logistic regression was used to estimate ORs and 95% CIs comparing the dense (BI-RADS = 3, 4) to non-dense breast group (BI-RADS=1, 2). Adjusted for province, age (per 2-year), family history, education, BMI (per 2 kg/m^2), age at menarche, parity, and menopausal status.

[b]Excluding women with first-degree relatives with breast cancer < 50 years.

[c]BMI (per 2 kg/m2) was replaced with height (per 2 cm) and weight (per 2 kg) in the model.

[d]Parous women only. Model was adjusted for province, age (2-year frequency), family history, education, BMI (per 2 kg/m2), age at menarche, age at first full term birth, breastfeeding, and menopausal status.
